# Knowledge regarding fertility preservation in cancer patients: a population-based survey among Brazilian people during the Pink October awareness event

**DOI:** 10.5935/1518-0557.20170021

**Published:** 2017

**Authors:** Mauricio B Chehin, Tatiana CS Bonetti, Paulo C Serafini, Eduardo LA Motta

**Affiliations:** 1 Huntington - Medicina Reprodutiva. Sao Paulo, SP, Brasil; 2 Disciplina de Ginecologia Endocrinológica, Departamento de Ginecologia, Escola Paulista de Medicina da Universidade Federal de São Paulo (UNIFESP-EPM). Sao Paulo, SP, Brasil; 3 Disciplina de Ginecologia, Departamento de Obstetrícia e Ginecologia, Faculdade de Medicina, Universidade de Sao Paulo (FMUSP). Sao Paulo, SP, Brasil

**Keywords:** oncofertility, cancer, fertility preservation, survey

## Abstract

**Objective:**

The aim of this study was to assess the knowledge about the risk of infertility in cancer patients after treatment, and the options for fertility preservation based on a survey carried out during the 2013 Pink October campaign.

**Methods:**

This survey was carried out during the 2013 Pink October event in the most important public park of São Paulo, Brazil. Approximately 900 people expressed interest in learning about breast cancer prevention and fertility preservation by participating in workshops, and 242 people filled out a questionnaire.

**Results:**

Most of the respondents (78.5%) were women, and one-fourth (25%) had at least one relative with gynecological cancer. Among women over 40 years of age, 86.3% had been screened for breast cancer at some point. However, few participants (34.0%) were aware that cancer treatment can lead to infertility or had heard about fertility preservation options (22.0%). Having a relative with cancer did not influence their knowledge about fertility preservation (22.4% versus 21.3%; *p*=0.864). However, a higher educational level was significantly associated with more knowledge about the effects of cancer on fertility and options for fertility preservation.

**Conclusions:**

The majority of participants did not have knowledge about the impact of oncologic treatment on fertility and did not know that there are options to preserve fertility in cancer patients. Awareness of infertility risk factors is an essential first step to safeguard future fertility, and therefore, more educational initiatives are needed to spread knowledge about oncofertility.

## INTRODUCTION

Advances in cancer therapy over the past two decades have led to a remarkable improvement in survival rates. Indeed, over the past 5 years, overall rates of death attributable to cancer in women have fallen by > 1.6% per year (^Siegel *et al.*, 2012^). In addition to increasing survival, addressing the diverse psychological and physical effects of malignancy is now a priority to optimize the quality-of-life of cancer survivors. The damaging effects of cancer treatments and, occasionally, of the disease process itself on reproductive function are well recognized (^Grynberg, 2016^). Loss of reproductive potential after cancer treatment represents an important issue for the patients' well-being, and it negatively affects quality-of-life in young survivors (^Tschudin & Bitzer, 2009^; ^Niemasik *et al.*, 2012^).

The concept of oncofertility was proposed a decade ago (^Woodruff, 2010^), and with the developments in assisted reproductive technologies, there are several fertility preservation approaches available for cancer patients wishing to have children in the future. Fertility is thus recognized as a critical component of quality-of-life for cancer survivors, and fertility preservation should be considered a natural extension of cancer care (^Rodriguez-Wallberg & Oktay, 2010^).

Although fertility should be at the forefront of cancer attention, and although international guidelines recommend that patients should be informed both of the risk of subsequent treatment-related infertility and the available options for fertility preservation (^Loren *et al.*, 2013^; ^Peccatori *et al.*, 2013^; ^Lambertini *et al.*, 2016^), oncologists often lack knowledge and fail to provide information to patients about infertility risks prior to the start of cancer treatment (^Anderson *et al.*, 2008^; ^Adams *et al.*, 2013^; ^Garrido-Colino *et al.*, 2016^; ^Ghazeeri *et al.*, 2016^; ^Warner *et al.*, 2016^). A survey performed by the France National Institute of Cancer demonstrated that a minimal percentage of female cancer patients (2.2%) had been informed about fertility preservation before treatment (^Peretti-Watel, 2014^). In practice, few cancer patients who are at risk of infertility are informed of these risks before treatment onset, and even fewer are referred to a specialist and offered options to preserve their fertility (^Anderson *et al.*, 2008^; ^Rodriguez-Wallberg & Oktay, 2010^; ^Corney & Swinglehurst, 2014^; ^Preaubert *et al.*, 2016^).

Breast cancer is the most common type of cancer in women, and the second most common type of cancer overall, with nearly 1.7 million new cases diagnosed in 2012 (^Ferlay *et al.*, 2013^). Breast cancer survival rates vary widely throughout the world, ranging from 80% or higher in North America, Sweden and Japan, to approximately 60% in middle-income countries, and below 40% in low-income nations (^Coleman *et al.*, 2008^). In less developed countries, a lack of early detection programs, adequate diagnosis and treatment facilities lead to low survival rates. The high prevalence of this disease prompted the development of a world-wide annual campaign in 1997, named Pink October, to increase awareness about Breast Cancer, which instituted October as the Breast Cancer Awareness Month.

Aiming to join this collaborative effort, the Huntington Reproductive Medicine group promoted a Pink October campaign in 2013, to inform patients about breast cancer prevention and fertility preservation in the major public park of São Paulo, Brazil. The event was open to everyone in the park (men and women), and approximately 900 people expressed interest in learning about breast cancer prevention and fertility preservation, participating in workshops and talking to fertility specialists. During this campaign, the participants were invited to answer a questionnaire about fertility preservation in cancer patients. The aim of this study was to assess the overall knowledge about the risks of infertility in cancer patients following oncologic treatment and the options for fertility preservation by utilizing a survey during the Huntington Reproductive Medicine Pink October campaign.

## MATERIALS AND METHODS

This was a qualitative study based on a survey about fertility preservation in cancer patients carried out during the 2013 Pink October event held in Ibirapuera park, São Paulo, Brazil. The instrument was an investigator-designed, self-reported questionnaire including a total of 24 questions about sociodemographic characteristics (6 questions), medical history (11 questions), life style (5 questions) and awareness concerning the subject of fertility preservation in cancer patients (3 questions). The participants who answered the questionnaire authorized the use of data in scientific publications, respecting the anonymized handling of data, according to the rules of ethics.

The questionnaires were available in paper-and-pencil format, and no patient identifiers were included to ensure anonymity. A total of 242 participants answered the questionnaire. The survey included questions on demographic characteristics, such as gender, age, educational status, occupation, and the following cancer-related questions: (1) Do you have a relative with gynecological cancer? and (2) Have you been previously screened for breast cancer? The survey also included questions on fertility preservation in cancer patients: (1) Have you heard about fertility preservation in cancer patients? (2) Do you know that patients can be infertile after cancer treatment? (3) Do you know anyone younger than 40 years who has had cancer?

### Statistical analysis

Descriptive statistics were used to characterize the population. To evaluate how much knowledge patients had about fertility preservation in cancer patients, their answers were presented as percentages. We used a Chi-square test to compare answers between subgroups and t-tests to compare continuous variables. The data was analyzed using the SPSS software (version 18, IBM, SPSS, Chicago, IL, USA).

## RESULTS

**Figure 1:**
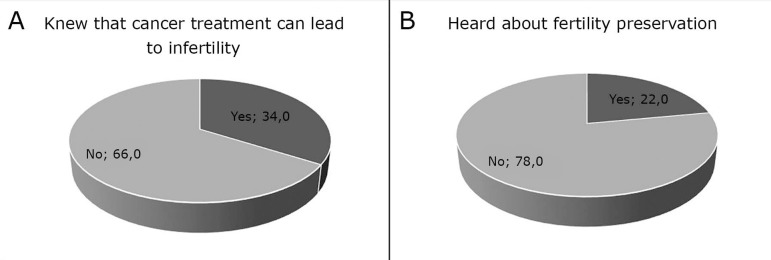
A: Percentage of participants who knew that cancer treatment can lead to infertility. B: Percentage of patients who had heard about fertility preservation options.

Two hundred forty-two people answered the survey; 78.5% (n = 190) were women (42.3±15.6 years of age), and 21.5% (n = 52) were men (45.5±17.7 years of age). Most of the participants had a college degree (65.9%), and one-fourth (24.6%) had a relative with gynecological cancer. Among the women who had relatives with a history of gynecological cancer, a higher percentage (82.7%) had been screened for breast cancer compared to those who did not have relatives with a history of gynecological cancer (64.4%, *p*=0.012). Among the 95 women over 40 years of age, independent of whether they had relatives with a history of gynecological cancer, 86.3% had previously been screened for breast cancer. However, only 34.0% of the participants were aware that cancer treatment can lead to infertility, and only 22.0% had heard about fertility preservation options (Figure 1A and 1B).

Knowledge about the risk of infertility following cancer treatment was similar between women and men (35.8% and 26.5%; *p*=0.214) and between those who had a relative with a history of gynecological cancer and those who did not (36.2% and 33.1%; *p*=0.670). Although there was no statistically significant difference, a higher percentage of women had heard about fertility preservation options for cancer patients (23.5%) when compared to men (14.3%; *p*=0.147), but the percentage was comparable between those who had relatives with a history of gynecological cancer (22.4%) and those who did not (21.3%; *p*=0.864).

**Figure 2:**
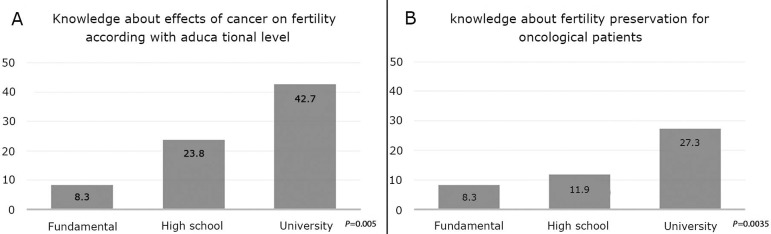
Graphical representations of the percentage of respondents who had knowledge about the effects of cancer treatment on fertility (A) and fertility preservation options for cancer patients (B).

Conversely, the level of education attained was positively correlated with both knowledge about the effects of cancer treatment on fertility and familiarity with fertility preservation options for cancer patients (Figure 2A and 2B). It is interesting to note that even among healthcare professionals, the knowledge was the same as that of the general population with a university-level education, in terms of the effects of cancer on fertility (11/24, 45.8%) and fertility preservation for cancer patients (6/24, 25.0%).

## DISCUSSION

Breast cancer is the most common malignancy in women (^Rojas & Stuckey, 2016^), and each year Pink October events spread awareness about prevention and treatment of this disease. Considering the impact of emotional impairment, relationship problems, uncertainty about maternity, safety and low self-esteem on the quality-of-life reported by young female cancer survivors (^Yee *et al.*, 2012^; ^Deshpande *et al.*, 2015^), fertility preservation brings hope and enhances self-competence in cancer treatment decisions. In this context, fertility has been identified as the second most important factor for cancer patients who are considering treatment options, surpassed only by survival (^Kim *et al.*, 2016^).

In contrast, our study found that only approximately one-third of people have information about fertility impairment after cancer treatment and that approximately 20% of the respondents were aware that fertility preservation is possible. Unexpectedly, people who had a relative with cancer did not have more knowledge about oncofertility, when compared to those who did not.

Previous studies demonstrated that the amount of information concerning oncofertility given to women by healthcare professionals varies considerably. Oncologists hold little knowledge about fertility preservation techniques, and the referral rates for oncofertility experts remain low (^Bastings *et al.*, 2014^; ^Corney & Swinglehurst, 2014^). The main reason for the low rate of oncofertility referrals is the complexity and novelty of some fertility preservation options, which include techniques such as ovarian tissue cryopreservation or oocyte *in vitro* maturation (^Donnez & Dolmans, 2013^), and thus make this discussion about reproductive health in young patients diagnosed with a fertility-threatening disease particularly complex. This discussion should cover topics from basic biology, medical practice planning, health access, and reproductive rights, and include ethical, social, moral, cultural, religious, and personal perspectives (^Woodruff, 2015^).

Our study also demonstrated that a higher educational level was positively correlated with knowledge about the effects of cancer treatment on fertility and about fertility preservation options for cancer patients. However, in line with the literature, it is interesting to note that healthcare professionals did not have more information about the effects of cancer or cancer treatment on fertility, or about fertility preservation options than the general population with a university-level education. This scenario suggests the need to spread information on oncofertility, as this is a new and complex field in medicine, that addresses the fertility preservation needs of young cancer patients, intersecting the disciplines of oncology and fertility (^Woodruff, 2007^).

Unfortunately, many patients stated that the discussion of fertility matters was initiated by themselves, their friends, and their families rather than their healthcare providers (Yee *et al.*, 2012), reinforcing the importance of educational initiatives to spread oncofertility concepts to healthcare professionals. In France, a study on an educational initiative to encourage physicians who were involved with treating cancer patients, to refer these patients for fertility consultations, reported an important and positive impact of providing the patient this information before starting treatment. Indeed, the percentage of physicians who always or often informed their patients about the risk of subsequent infertility prior to potentially gonadotoxic treatments increased from 53.8% to 81.0%, and the percentage of physicians who informed their patients about fertility preservation options increased from 44.9% to 75.7% after three years of the program (^Preaubert *et al.*, 2016^).

Furthermore, several society-based guidelines recommend the discussion of potential fertility impairment at the earliest possible moment after diagnosis, a prompt referral to a qualified specialist if the patient is interested, and the promotion of clinical trials to advance the state of art in fertility preservation (^Ethics Committee of American Society for Reproductive, 2013^; ^Loren *et al.*, 2013^). Currently, sperm, egg, and embryo banking are standards-of-care for preserving fertility for cancer patients at reproductive age; ovarian tissue cryopreservation is still considered experimental (^Kim *et al.*, 2016^). Our group has been vitrifying oocytes from cancer patients since it was an experimental procedure (^da Motta *et al.*, 2014^) and has worked hard to inform healthcare professionals about oncofertility. It is necessary that the medical community acquire knowledge about these issues to inform and refer young cancer patients in an efficient way for fertility preservation.

Making decisions about preserving future fertility requires that patients receive information from many different sources, including their doctors, their families, and society, at a highly emotional time, when they are facing an existential crisis about themselves, their survival, and their future. This survey reveals the lack of oncofertility knowledge among the Brazilian population and supports advocating for a healthcare field that bridges oncology and fertility to discuss reproduction with young cancer survivors. The Brazilian Oncofertility Consortium has advocated for oncofertility concerns in the last several years, not only in the country itself but also working with global partners in the scientific field around the world (^Ataman *et al.*, 2016^). Valuing the Brazilian oncofertility community, it is important to spread the knowledge about this subject among oncologists, general gynecologists, urologists and others, and this will consequently lead to better fertility support for young, childless cancer patients.
